# Recommendations for improving accuracy of gene expression data in bone and cartilage tissue engineering

**DOI:** 10.1038/s41598-018-33242-z

**Published:** 2018-10-05

**Authors:** Tao He, Yijiang Huang, Juy Chi Chak, Roland Manfred Klar

**Affiliations:** 1 Laboratory of Biomechanics and Experimental Orthopaedics, Department of Orthopaedic Surgery, Physical Medicine and Rehabilitation, University Hospital of Munich (LMU), Munich, Germany; 2 Department of Orthopaedics, Renji Hospital, School of Medicine, Shanghai Jiaotong University, Shanghai, China

## Abstract

Autogenous tissue grafting remains the gold standard in the treatment of critical sized bone and certain cartilage defects, while the translation of tissue engineered osteogenesis or chondrogenesis from the lab bench into clinical practice, utilizing natural or synthetic biomimetic devices, remains challenging. One of the crucial underestimated reasons for non-translatability could be the imprecision and inconsistency of generated gene expression profiles, utilizing improperly optimized and standardized quantitative gene assays. Utilizing GeNorm for downstream qRT-PCR applications, the stability of reference genes in relation to optimal cDNA amounts was assessed on human bone marrow-derived mesenchymal and adipose-derived stem cells neat and made to differentiate into chondrocytes including normal human derived chondrocytes and muscle tissue from rats. Results showed that reference genes can vary substantially across separately and/or combined cell lines and/or tissue types including treatment parameters. The recommendations to all bone and cartilage tissue engineers utilizing qRT-PCR is not to assume that reference gene stability and quantity remain conserved across cell lines or tissue types but to always determine, for each new experiment, the stability and normalization quantity of reference genes anew.

## Introduction

After more than a century, clinical bone regenerative procedures still rely on autogenous bone grafting^1–3^ to heal and regenerate large bone defects in human. Similarly, damaged cartilage caused either by osteoarthritis or intensive sports are still being treated by varying surgically invasive procedures with limited regenerative techniques being unable to heal critical defects^4–9^. Whilst some progress has been made in our understanding in some of the molecular mechanisms and how bone and cartilage formation can be induced experimentally the concept of either regenerating defects or complete re-growth of the damaged osteogenic or chondrogenic material remains problematic^10–20^. One factor not often considered is the variety of analytical methodologies used to monitor gene expression patterns and their subsequent translation, which are deciding factors for generating realistic mechanistic molecular insights in how regenerative processes function and how to modulate them.

Real-time reverse transcription quantitative polymerase chain reaction (qRT-PCR) has become the leading analytic technique, and is an economical, jet simple and highly effective method for monitoring gene transcription, which over the last two decades has made considerable progress at improving the accuracy of gene expression data^21–31^. Quantitative real-time PCR remains the best alternative to new emerging techniques such as digital droplet PCR and Next Generation Sequencing that are often very expensive to perform. Whilst some bone and cartilage research groups have already used the new standards as set out by Bustin *et al*.^27^ pioneering “Minimum Information for Publication of Quantitative Real-Time PCR Experiments (MIQE) guidelines”^20,27,32–34^, a considerable number of research groups still rely on outdated and highly insufficient qRT-PCR techniques; when combined with insufficient experimental detail, these techniques render replication of many published findings challenging^35^ and questionable. The lack of accurate and reproducible gene expression data could be one of the factors contributing to the failing or weak translatability of the *in vitro* to *in vivo* to the clinical context, thereby preventing regenerative therapies from replacing the gold standards currently employed to treat bone and cartilage damage and defects.

## Results

### cDNA amount dependent reference genes expression

In all cell lines and tissue included in the present study, the mean Cq of *RNA28S* was the lowest (Table 1). For human bone-derived mesenchymal stem cells (hBMSCs), the mean Cq of *RPL13a* was consistent with complementary DNA (cDNA) quantity ranging from 2.5 ng to 40 ng (20.03 ± 0.68); the mean Cq of *SDHA*, *TBP*, *RNA28S4*, *GAPDH*, and *RPLP0* decreased as the amount of cDNA increased; the mean Cq of *POLR2e* and *ACTB* increased as the amount of cDNA increased (Table 1). For human adipose-derived mesenchymal stem cells (hADSCs) and human chondrocytes, the mean threshold cycle of all eight candidate reference genes decreased as the amount of cDNA increased. The two reference genes with the highest mean Cq were *SDHA* and *TBP* (Table 1). Interestingly, for the rat rectus abdominis muscle, the mean threshold cycle of *TBP* and *GAPDH* remained constant independently of the cDNA quantity utilized (26.46 ± 0.96, and 26.64 ± 0.24 respectively); the mean Cq of *POLR2e* and *RNA28S4* decreased as the amount of cDNA increased for the muscle tissue with the mean Cq of *SDHA*, *ACTB*, *RPL13a*, and *RPLP0* increasing as the amount of cDNA increased (Table 1). Optimum cDNA quantity, to use for all other qRT-PCR applications, was determined to be between 2.5 ng to 10 ng which permits for maximum usage of reference genes and test genes to be used during qRT-PCR assays without waste of RNA or subsequent cDNA.

**Table 1:** Mean threshold cycle (Cq) of reference genes in a 2x dilution series of cDNA quantities from chondrogenic hMSCs (Chond.), hADSCs (Chond.), normal human chondrocytes (hChond.) and muscle tissue from rat.

Reference Gene	Cell/Tissue Type	Cq at cDNA quantity				
		2.5 ng	5 ng	10 ng	20 ng	40 ng
*SDHA*	hMSCs (Chond.)	25.52	24.45	23.72	22.94	22.66
	hADSCs (Chond.)	27.07	26.09	24.98	24.03	23.07
	hChond.	26.94	25.82	24.63	23.51	22.74
	Muscle tissue (rat)	22.63	21.51	20.77	21.18	25.89
*POLR2e*	hMSCs (Chond.)	25.13	23.90	23.03	22.21	29.57
	hADSCs (Chond.)	27.75	26.37	25.49	24.42	24.23
	hChond.	23.60	22.98	21.38	20.42	19.88
	Muscle tissue (rat)	23.91	22.92	22.34	21.61	22.35
*TBP*	hMSCs (Chond.)	28.02	26.73	26.02	25.34	25.77
	hADSCs (Chond.)	28.07	27.10	26.10	25.21	24.04
	hChond.	26.06	25.09	24.05	22.91	21.9
	Muscle tissue (rat)	27.40	26.44	25.70	25.33	27.43
*ACTB*	hMSCs (Chond.)	17.10	16.02	15.48	16.51	23.83
	hADSCs (Chond.)	25.45	23.96	22.73	21.81	21.19
	hChond.	16.94	15.83	14.86	13.82	12.80
	Muscle tissue (rat)	22.26	21.07	20.93	20.89	23.59
*RPL13a*	hMSCs (Chond.)	20.72	19.88	20.22	18.94	20.39
	hADSCs (Chond.)	18.26	17.03	15.90	14.99	13.85
	hChond.	19.83	18.71	17.45	16.68	15.82
	Muscle tissue (rat)	20.83	20.10	20.04	19.99	22.18
*RNA28S4*	hMSCs (Chond.)	12.63	11.38	10.63	9.76	8.93
	hADSCs (Chond.)	13.95	12.84	11.93	10.82	11.30
	hChond.	12.70	11.70	10.62	9.81	8.98
	Muscle tissue (rat)	12.60	11.63	10.26	8.93	8.98
*GAPDH*	hMSCs (Chond.)	19.48	18.38	17.62	17.07	16.66
	hADSCs (Chond.)	23.04	22.02	20.55	19.60	18.53
	hChond.	18.31	17.12	16.02	15.08	14.10
	Muscle tissue (rat)	26.78	26.49	26.31	26.93	26.69
*RPLP0*	hMSCs (Chond.)	20.70	19.56	19.06	18.59	18.75
	hADSCs (Chond.)	25.32	24.07	22.87	21.93	21.39
	hChond	19.07	17.26	16.37	15.40	14.81
	Muscle tissue (rat)	26.80	25.99	25.77	26.35	29.82

### Stability and optimal number of reference gene(s) expression

Cells were assessed separately, in combination for a single cell or tissue type, differentiated or undifferentiated, or as a total combination of all cells and tissue types. The different analyses represent different hypothetical experimental scenarios that could come about during a study. Scenarios are divided into: hBMSCs normal and differentiated into chondrocytes; hADSCs normal and differentiated into chondrocytes; normal chondrocytes and normal chondrocytes treated (made apoptotic); hADSCs/differentiated hADSCs with hBMSCs/differentiated hBMSCs with normal/treated chondrocytes with the final scenario being rat muscle tissue normal and osteogenic medium treatment.

#### hBMSCs and chondrogenic hBMSCs

**Figure 1:**
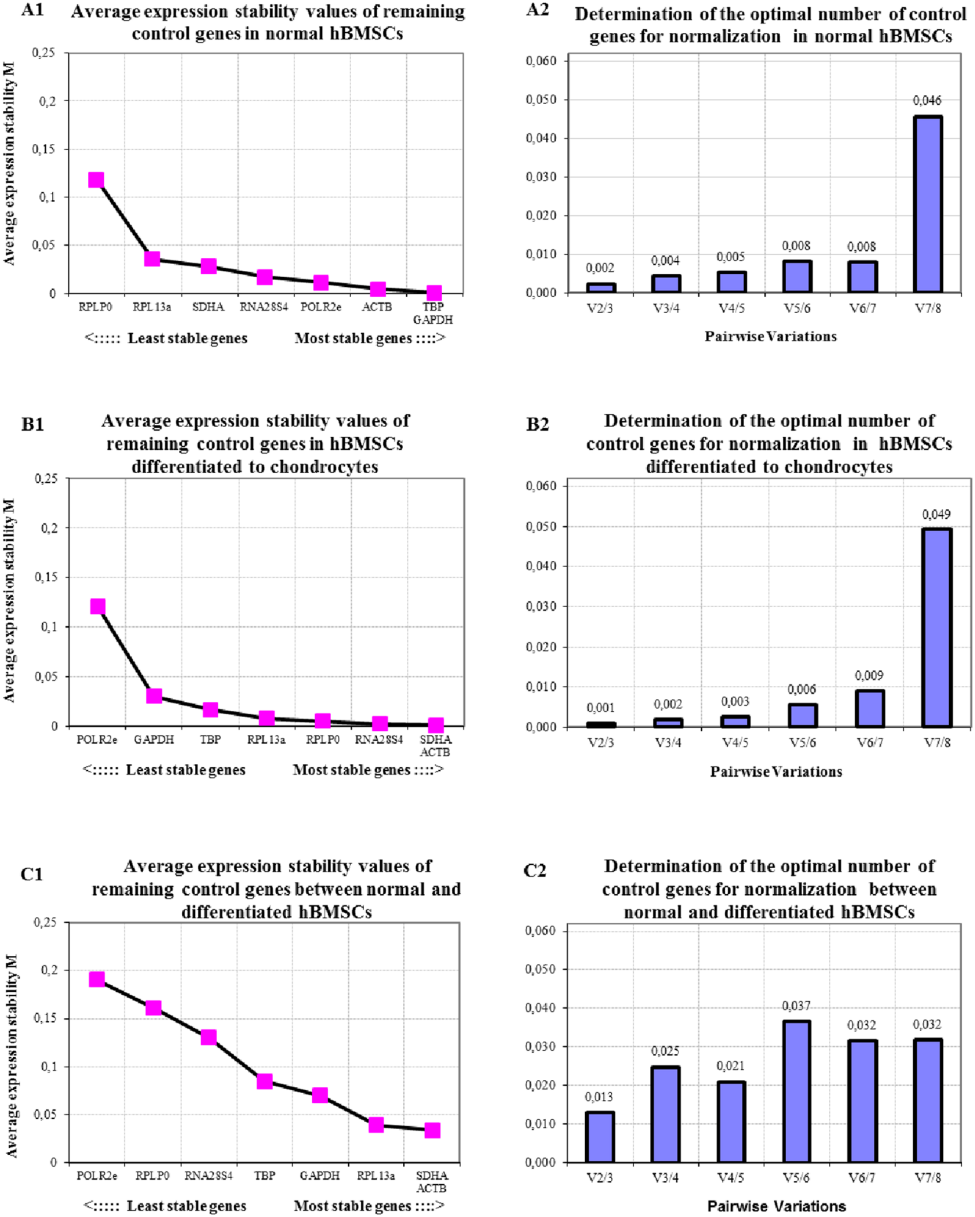
Average expression stability (**A1**,**B1**,**C1**) and optimal number of reference genes for normalization (**A2**,**B2**,**C2**) for qRT-PCR assays, utilizing the GeNorm algorithm, for (**A**) normal hBMSCs and (**B**) chondrogenic differentiated hBMSCs separately or (**C**) both cell lines combined.

GeNorm analysis was carried out singly for hBMSCs and hBMSCs differentiated into chondrocytes including both types of cell types combined (Fig. 1A–C). For undifferentiated hBMSCs the most stable expressed reference primers were *TBP*, *GAPDH*, *ACTB* and *POLR2e* (Fig. 1A1), with *SDHA*, *ACTB*, *RNA28S4* and *RPLP0* being the reference genes with the best stability in hBMSCs that had undergone chondrogenic differentiation (Fig. 1B1). When both cell types were assessed in combination, using GeNorm, *SDHA*, *ACTB*, *RPL13a* and *GAPDH* were most stably expressed (Fig. 1C1). With respect to the optimal number of reference genes necessary for normalization, results showed that 2 to 3 reference genes were sufficient for both single and combined GeNorm analysis sets (Fig. 1A2–C2).

#### hADSCs and chondrogenic hADSCs

**Figure 2:**
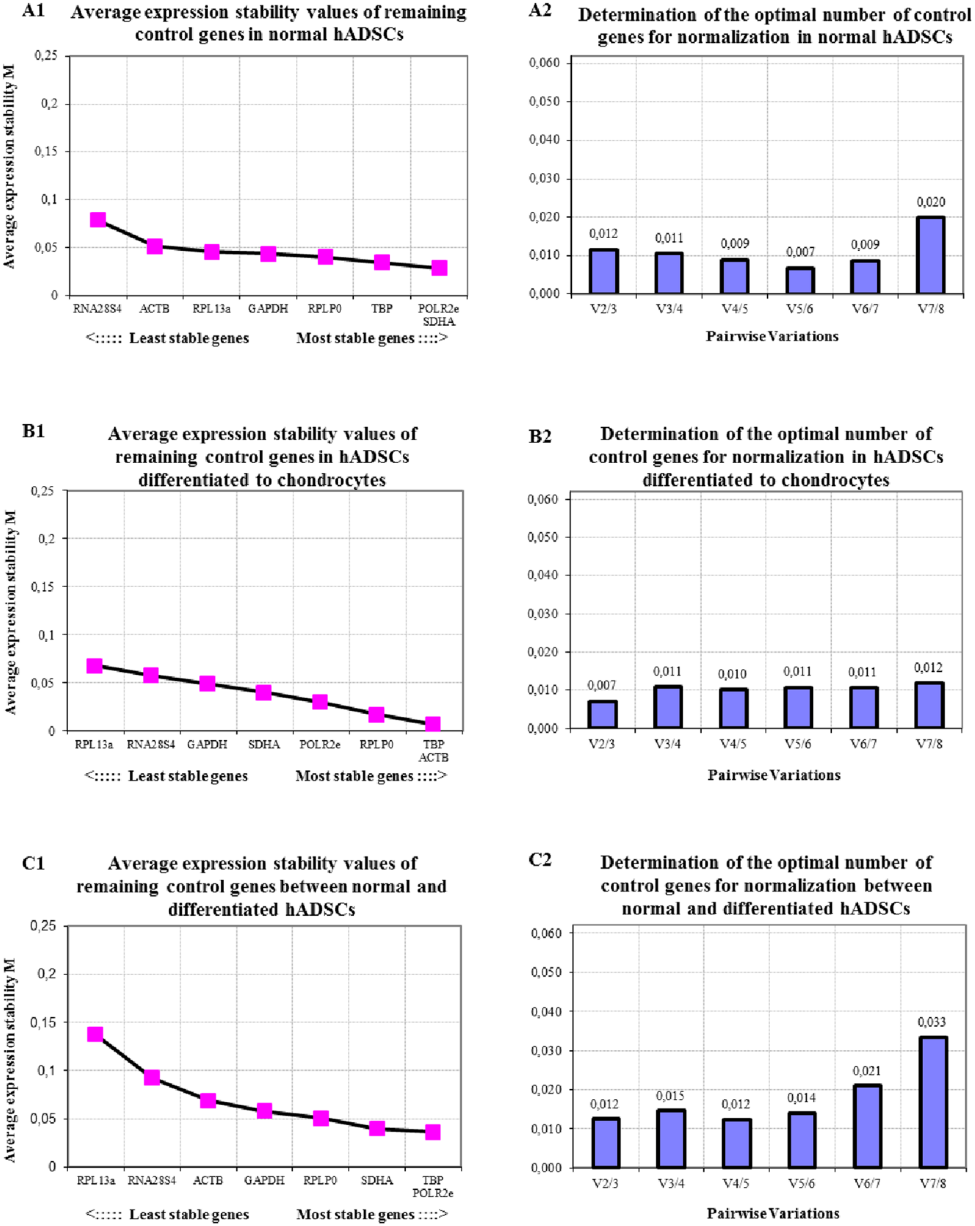
Average expression stability (**A1**,**B1**,**C1**) and optimal number of reference genes for normalization (**A2**,**B2**,**C2**) for qRT-PCR assays, utilizing the GeNorm algorithm, for (**A**) normal hADSCs and (**B**) chondrogenic differentiated hADSCs separately or (**C**) both untreated and treated hADSCs cell lines combined.

As shown in Fig. 2, the optimal number of reference genes to use for normalization during a qRT-PCR analysis was 5 to 6 for the hADSCs group singly, 2 to 3 in chondrogenic hADSCs singly and 4 to 5 when both hADSCs normal/differentiated were analyzed together utilizing GeNorm, respectively (Fig. 2A2–C2). The reference gene expression stability of untreated hADSCs, chondrogenic hADSCs or combined GeNorm analysis sets generated similar results, in which *TBP*, *SDHA*, *POLR2e*, *RPLP0* and *ACTB*/*GAPDH* were among the most stable reference genes expressed (Fig. 2A1–C1). The order of these reference genes on the stability scale changed. In general, the highest six reference genes in the ranking of stability were *POLR2e*, *SDHA*, *TBP*, *RPLP0*, *GAPDH*, and *RPL13a* in untreated hADSCs; *TBP*, *ACTB*, *RPLP0*, *POLR2e*, *SDHA*, and *GAPDH* in chondrogenic hADSCs; *TBP*, *POLR2e*, *SDHA*, *RPLP0*, *GAPDH*, and *ACTB* in combined hADSCs sets.

#### Normal and treated (apoptotic) chondrocytes

**Figure 3:**
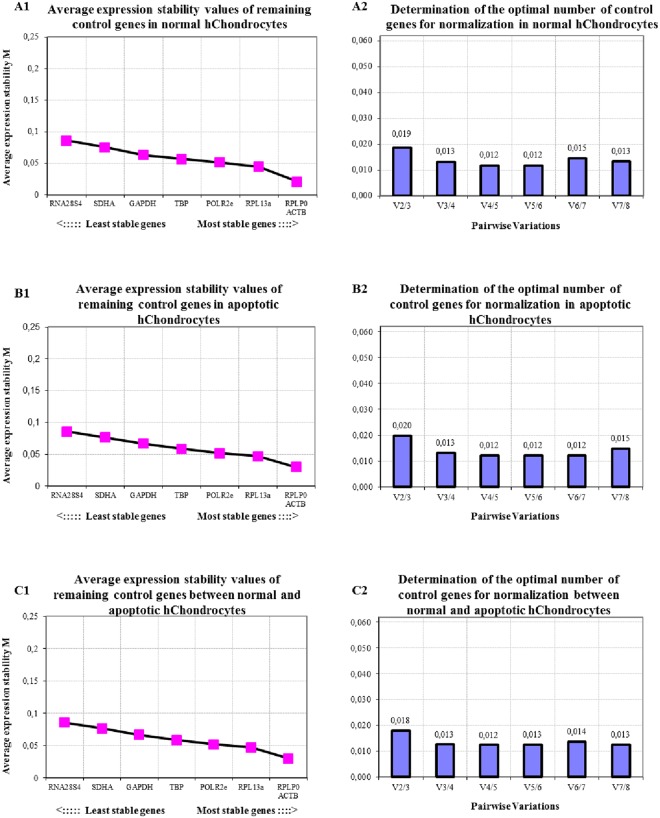
Average expression stability (**A1**,**B1**,**C1**) and optimal number of reference genes for normalization (**A2**,**B2**,**C2**) for qRT-PCR assays, utilizing the GeNorm algorithm, for (**A**) normal human Chondrocytes (hChondrocytes) and (**B**) hChondrocytes undergoing apoptosis or (**C**) both normal and apoptotic chondrocyte cell lines combined.

ATDC5 human chondrocytes untreated or made apoptotic (treatment), show that the optimal number of reference genes to use for normalization in qRT-PCR, after GeNorm assessment, is 5 to 6 for untreated chondrocytes, 6 to 7 for the treated chondrocyte group and 4 to 5 when both untreated and apoptotic chondrocyte groups are assed together (Fig. 3A2–C2). The reference gene expression stability order between the different groups remained consistent (Fig. 3A1–C1). The highest seven reference genes in the ranking of stability from most to least stable were *RPLP0*, *ACTB*, *RPL13a*, *POLR2e*, *TBP*, *GAPDH*, and *SDHA*.

#### Chondrocytes, hBMSCs and hADSCs

**Figure 4:**
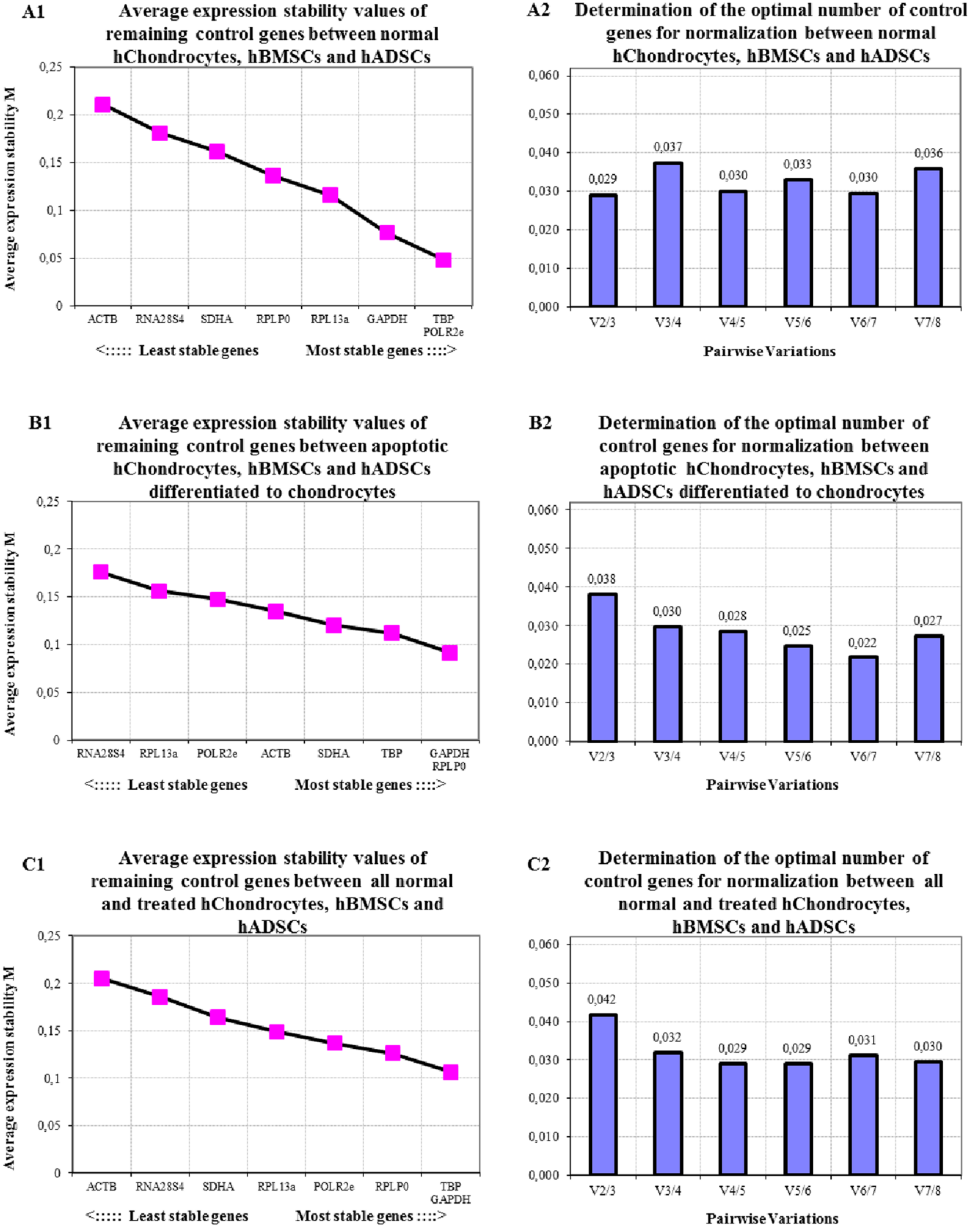
Average expression stability (**A1**,**B1**,**C1**) and optimal number of reference genes for normalization (**A2**,**B2**,**C2**) for qRT-PCR assays, utilizing the GeNorm algorithm. (**A**) GeNorm results between Control cell groups with normal hChondrocytes, hBMSCs and hADSCs. (**B**) GeNorm results between treated cell groups, i.e. hChondrocytes undergoing apoptosis, chondrogenic differentiated hBMSCs and hADSCs. (**C**) GeNorm results between all untreated and treated cell lines and types.

For all normal, treated or differentiated cell types, results showed that the optimal number of reference genes to use for normalization during a qRT-PCR analysis are 2 to 3 when chondrocytes, hBMSCs and hADSCs were looked at in GeNorm, 6 to 7 when treated chondrocytes (apoptotic), chondrogenic hBMSCs and chondrogenic hADSCs were assessed with GeNorm and 5 to 6 when all cell groups were assessed, respectively (Fig. 4A2–C2). For reference gene expression stability between these different scenario groups *TBP*, *POLR2e*, *GAPDH* and *RPL13a* were the most stable genes in the chondrocyte, hBMSCs and hADSCs group, *GAPDH*, *RPLP0*, *TBP* and *SDHA* were the most stable in treated chondrocytes, chondrogenic hBMSCs and hADSCs with *TBP*, *GAPDH*, *RPLP0* and *POLR2e* being the most stable between all cell groups (Fig. 4A1–C1).

#### Rattus novericus rectus abdominis muscle normal and osteogenic

**Figure 5:**
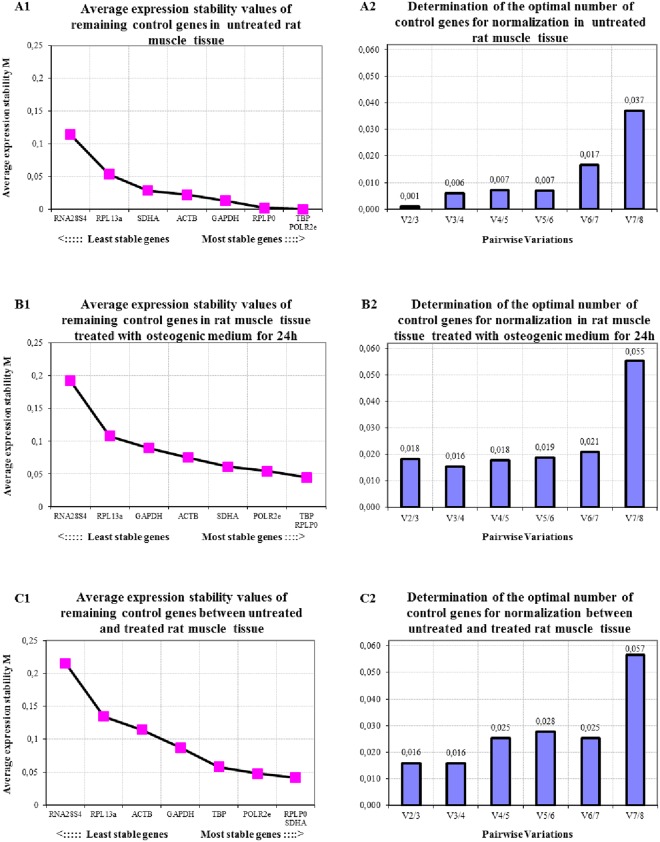
Average expression stability (**A1**,**B1**,**C1**) and optimal number of reference genes for normalization (**A2**,**B2**,**C2**) for qRT-PCR assays, utilizing the GeNorm algorithm, for (**A**) untreated rat *rectus abdominis* muscle tissue, (**B**) rat *rectus abdominis* muscle tissue treated with osteogenic medium, and (**C**) both normal and treated rat muscle tissue.

Untreated muscle tissue, including muscle tissue that had been stimulated to undergo osteogenic differentiation utilizing osteogenic medium within an *in vitro* culture system, were analyzed separately and combined using GeNorm assessment. Results show that that reference stability changes considerably when untreated, treated and combined experimental groups were assessed (Fig. 5A1–C1), where ranking of the reference gene is concerned on the stability curve. The highest four most stable reference genes in untreated rat muscle were *TBP*, *POLR2e*, *RPLP0*, and *GAPDH*. *TBP*, *RPLP0*, *POLR2e*, and *SDHA* were most stable in the osteogenic treated rat muscle group, whereas *RPLP0*, *SDHA*, *POLR2e*, and *TBP* were in combined rat muscle groups. As for the optimal number of reference genes needed for normalization, the results show that 2 to 3 are needed in untreated rat muscle group with 3 to 4 in osteogenic rat muscle or both untreated and treatment groups combined (Fig. 5A2–C2). The overall gene expression stability of untreated rat muscle was the highest, while osteogenic rat muscle ranked second with the combined groups coming last.

## Discussion

The results from the present study clearly show that reference genes in many relative qRT-PCR applications are inappropriately used, as the housekeeping gene expression changes between cell and tissue types including how these are utilized under various experimental conditions. Reference gene(s) stability varies together with the appropriate number of candidate reference genes to use during qRT-PCR gene expression analyses. This can significantly alter the gene expression results and subsequent downstream interpretations, consequently reducing the accuracy of gene transcription patterns that are necessary to make appropriate deductions for molecular mechanisms in tissue engineering fields.

Regeneration of bone and cartilage tissue without the need for autografting is a promising aspect in medicine. However, the translation from *in vitro* to *in vivo* and finally into the clinical setting remains problematic and challenging^36^. Results from numerous studies are difficult to interpret or compare with each other as there is a considerable heterogeneity in experimental designs. Whether it is different stem cell or cell lines, tissue types, varying time points of analysis during experimental setups or the plethora of different treatments, there is currently no standardized concept in the field of bone and cartilage tissue engineering in the way of methodologies. This however is a crucial criterion to ensure that generated data remains consistent and reproducible. One critical aspect in the “standardization debate” is the generation of accurate gene expression data, as genes control life. Understanding the patterns of modulation is critical to successfully regenerate bone, cartilage or any tissue type *in vivo*^37^. However, few groups do conform to new standards in qRT-PCR applications. The use of validated techniques and the quantity of reference genes for normalization remains problematic but are an important criterion without which, the generation of accurate, comparable and reproducible gene expression results cannot be obtained, thereby making the generated genetic expression profile of cells or tissue during bone or cartilage regenerative procedures results questionable. Whilst more modern techniques exist, they are prohibitively expensive but at the same time require validation through qRT-PCR assays, to determine the validity of generated results^38,39^. As such, if used properly, qRT-PCR remains the best alternative. As of now, variation still exists as few studies conform to the new and proven standards by Bustin, Hellemans and Vandesompele^27–31^. This might explain why bone and cartilage regeneration still appear to fail clinically. The present study attempted to optimize and validate necessary reference genes for specific cell and tissue types that are most commonly used in bone and cartilage tissue engineering studies. What we discovered was a critical aspect in the validation process of reference genes to utilize during qRT-PCR experiments and generating the corresponding relative quantitative gene expression data of test genes, which has to be considered to ensure that future gene expression results would become more exact, but more importantly comparable and reproducible.

Reference genes are considered to be genes that are constantly expressed by cells, individually or or within tissue^40^. Various studies have shown that this is not to be taken as a rule as these genes can also be affected by treatment modalities^41^.The recommendations by Bustin *et al*.^27^ are to use a minimum of at least 2 reference genes during a qRT-PCR run, as the original reference gene of choice could be affected depending on the experiment. Our study clearly re-iterated this fact for the *GAPDH* gene, a very common utilized reference gene in bone and cartilage qRT-PCR assays^42–44^. This gene is considered to be the most “stably” expressed reference gene, with many presented experimental publications and groups never deviating or questioning the validity of this concept. However, from the results of the present study it is clear that whilst *GAPDH* can be one of the stably expressed reference genes, its position on the stability scale can greatly deviate. This, depending on the quantity of reference genes necessary for normalization, can considerably alter the gene expression profile of selected analytical genes that an experiment is trying to determine.

GeNorm^25^, NormFinder^45^, and BestKeeper^46^ are currently the three most used algorithms for determining the gene expression stability and the quantity of reference genes to use for normalization in qRT-PCR. Given that the GeNorm algorithm is highly dependent on the assumption that the reference genes being analyzed are not co-regulated^45^, in the present study an analysis to judge whether co-regulation existed between reference genes, was conducted. This way, it can be predicted that if two genes indeed co-regulated each other, when analyzed with GeNorm, they would occupy adjacent positions in the ranking^47^. As such an internal control was performed where different reference genes were removed (data not shown). Results validated that the ranking of reference genes related to stability and optimal number was not altered, meaning that there was no co-regulation between the different genes selected. These findings are supported by previous results where NormFinder and BestKeeper algorithms were utilized instead of GeNorm^48^. Although there have been suggestions to use both GeNorm in conjunction with BestKeeper or NormFinder or to only use BestKeeper with NormFinder^45–47^, the present results clearly indicate that irrelevant of the gene stability and quantity determining reference gene programs utilized, these will not impact on which reference genes are stable or the optimum number to use in an experiment. The GeNorm algorithm can be considered a reliable and very simple method for analyzing gene expression stability and determining the optimal number of reference genes for a selected study. Yet, what is of critical importance is to not assume that, once a certain reference gene set has been determined for a cell or tissue type within an experiment, such reference genes will always remain stably expressed, when utilizing this same cell or tissue type in consecutive experiments. This notion of using reference genes like this^49–51^ is dangerous and should be evaluated carefully in light of the present study’s findings.

Conducting qRT-PCR experiments in light of the new recommendations by Bustin *et al*.^31^ sometimes relies on optimized kits from various scientific companies or reference genes based on the recommendations from other research groups^50–53^. Care must however be made regarding the methodological steps present in the current or past literature, as these are often not detailed enough to allow reproducing experiments. This is particularly critical when reporting how the qRT-PCR sections are constructed. A good example, for the tissue engineering bone and cartilage fields are the publications by Klar *et al*.^32^, Klar *et al*.^20^, Ripamonti *et al*.^54^, where the qRT-PCR sections describe in great detail each step and how the material was optimized^20,32,54^. If publications do not offer this amount of detail, then the material must be carefully considered before use. This is made very clear when comparing the GeNorm results, from the present study, to the literature where similar designed studies utilizing similar cell types make recommendations to reference gene usage in qRT-PCR based experiments^55^. Interestingly, we discovered that even for a certain cell line with slightly different cell sources, for instance isolated cells from human tissue directly *versus* commercially available cell lines, results were considerably different. Compared to the study by Ragni *et al*.^55^, in which the study also included *TBP*, *ACTB*, *RPLP0*, *GAPDH*, and *RPL13A* as the candidate reference genes for chondrogenic human stem cells, the optimal number of reference genes to use was 9 to10 in their study, whereas our results suggested a minimum of 4 to a maximum of 5. Subsequently to this, the ranking of the reference genes expression stability also varied considerably between these two datasets with the sequence from most stable to least stable reference genes being *TBP*, *RPL13*A, *GAPDH*, *RPLP0* and *ACTB* from the chosen literature source, whereas in the present studies results it was *ACTB*, *RPLP0*, *GAPDH*, *RPL13A* and *TBP*. Furthermore, we found that when we put all the different cell groups together, be they untreated or treated groups, and assessed these in GeNorm analysis (Fig. 4C1,C2), the generated results of the reference gene expression stability *versus* optimal number of candidate reference genes to use for qRT-PCR applications varied substantially from those where only untreated or treated samples were assessed with each other separately (Fig. 4A1,A2,B1,B2). With respect to inductive tissues used for bone or cartilage regeneration, there are also considerable differences between *in vitro versus in vivo* based tissue gene expression patterns^51,56–59^. For the *rectus abdominis* muscle tissue from rats, since the tissue is comprised of a plethora of different cell layers and cell types, the stability of gene expression could be compensated for (Fig. 5), which is not the case for cells *in vitro* as these are often single cell lines lacking the presence of other cell types. However, similar to the patterns shown in cell lines of the present study, decreased stability of reference gene expression was also observed in the tissue when both untreated and treatment samples were combined in the GeNorm analysis. Similar to cells *in vitro*, there seems to be a pattern in the GeNorm analysis, where the moment the various groups are co-analyzed, the amount of reference genes to use to for the normalization varies. As such, it can be implied that subtle differences between different datasets can produce considerable variations in the gene expression stability and the optimal number of reference genes. From this, we thus make the recommendation not to assume that reference gene stability and quantity remains conserved for cell lines or tissue types between experiments but that there is a need to determine, for each new experiment, the stability and normalization quantity of reference genes anew. We believe that failing to do so might result in deviations in gene expression accuracy patterns during downstream analytical processes and consequently lead to difficulty in the translation of approaches to a clinical environment.

Of course, it must be said that the reference genes used in the current study are not all the reference genes presently available. There are a variety of reference genes produced by cells in addition to the 8 selected for this study^60^. As such we cannot definitively state that the presented reference genes are the most stable reference genes for cell types or tissue types in bone or cartilage research. To compensate for this, digital droplet PCR or Next Generation Sequencing could be utilized as these techniques eliminate the necessity for utilizing reference genes. However, since such technologies for gene analysis are very expensive to acquire and perform^38,39^, if normal qRT-PCR is used, it appears crucial that studies account for all know reference genes and be based on reference primers that are optimized specifically for each experiment rather than relying on risky assumptions that reference genes used in a similar study also be adequate for this specific experiment.

A subsequent important limitation is RNA quality. This could be one of the reasons why our results in terms of stability and normalization quantity deviates from other groups results^55,61,62^. Research has established that there is a direct link between quality of RNA and qRT-PCR data. Is the quality poor, results can deviate considerably in gene expression patterns^63^. In the present study, we utilized a modified Trizol extraction procedure^64^ out of economic reasons, whose outcomes in RNA quantities are user-dependent. Since the quality of RNA was good for the present study with RINs being between 6 to 8, the variation in quality cannot as such be based on the method but rather the user who is doing the extraction^65^. This is a further limitation that possibly prevented consistency in generated results with other groups^66,67^. Four different users performed the method separately, for each of the cell lines and the tissue type, as these results are part of other experiments. We believe that more accurate data might have emerged from experiments conducted by a single user, and might have yielded results that would be similar to literature^55,61,62,68^.

In conclusion, qRT- PCR remains the best validated technique to economically generate accurate relative gene expression results, which, when properly standardized, are required to develop effective treatments for healing bone or cartilage defects. Attempts at translating from cell to animal and ultimately to human in most cases are probably failing because of inaccurate gene expression results from outsourced and non-viable qRT-PCR methods, as these require a lot of pre-optimizations. This is supported by the present results. However, reference genes are also genes and can be affected by experimental conditions. We therefore suggest not to use reference genes from other study groups, even if the same cell line or tissue type is utilized, as the reference genes can differ between experiments which can lead to inaccuracies in gene expression results. Taken in account together with the RNA quality, we recommend that for all future studies involving qRT-PCR, in bone and cartilage research, laboratories should ensure that their RNA quality is consistently good and before doing qRT-PCR according the guidelines of Bustin *et al*.^31^, determine which reference genes are suitable for their given experiments. This muct be done by utilizing all known reference genes and defining which are the most stable and how many reference genes are necessary to generate accurate results. Future research can then establish a general standard, with respect to stable reference genes for cell and tissue types in bone and cartilage regenerative research. This would enable future scientists in the field to always generate the most consistent, accurate and reproducible gene expression data. If these steps are not performed, we fear that bone and cartilage regeneration will remain problematic clinically.

## Materials and Methods

### Cell and Tissue Specimens

Cell and tissue types chosen are commonly utilized for studying chondrogenic or osteogenic differentiation or regeneration. Human ADSCs, hBMSCs, chondrocytes and muscle tissue from *Rattus novericus* where utilized in this optimization and standardization study. A total of 12 specimens per cell and tissue type were utilized with 6 specimens acting as the untreated cell or tissue group with the remaining 6 specimens being treated to either undergo chondrogenic differentiation (all stem cells) or osteogenic differentiation (tissue type). For hADSCs, cells were donated to our lab by Maryna *et al*.^69^, whereas chondrocytes and hBMSCs were obtained from Lonza (Lonza, Basel, Switzerland).

*Rattus novericus rectus abdomins* muscle tissue was obtained by killing a F-344 adult male rat (Charles River Sulzbach, Germany) with an overdose of isoflurane (Abbot, Chicago, U.S.A.). This was done in accordance to the rules and regulations of the Animal Protection Laboratory Animal Regulations (2013), European Directive 2010/63/EU and approved by the Animal ethics research committee (AESC) of the Ludwig Maximillian’s University of Munich (LMU), Bavaria, Germany Tierschutzgesetz §1/§4/§17 (https://www.gesetze-im-internet.de/tierschg/TierSchG.pdf) with respect to animal usage for pure tissue or organ harvest only. Utilising 4 mm dermal biopsy punches (PFM medical, Cologne, Germany), 12 muscle tissue fragments were harvested. Six of the muscle fragments were treated to undergo osteogenesis utilising osteogenic medium (treatment) for 24 h, with the remaining six tissue fragments left untreated in DMEM. All tissue specimens were subsequently flash frozen in liquid nitrogen after which they were stored at −80 °C to be used for downstream molecular applications.

### Chondrogenic differentiation

Human BMSCs and hADSCs were stimulated to undergo chondrogenic differentiation, to determine if reference genes remain the same within and between the two stem cell types or are differentially expressed. Briefly, after stem cells had entered the fourth passage, stem cell cultures were supplemented with chondrogenic differentiation medium comprised of DMEM–high glucose (DMEM-hg), 10% fetal bovine serum, 100 units/ml penicillin, and 100 mg/ml streptomycin, 1x insulin-transferrin-selenium supplement (ITS + 1, Sigma-Aldrich), 50 µg/ml ascorbate 2-phosphate (Sigma-Aldrich), 40 µg/ml L-proline (Sigma-Aldrich),100 nm dexamethasone (Sigma-Aldrich), and 10 ng/ml rhTGF-β_3_ (R&D Systems, Minneapolis, U.S.A.). Every two days medium was changed, and cells cultured for 14 days to permit for differentiation. Chondrogenic differentiation was then confirmed via *ACAN* gene marker test, which is expressed only by differentiated chondrocytes. Differentiated cells as well as untreated control cells were then flash frozen in liquid nitrogen to be used for downstream RNA and qRT-PCR standardisation protocols.

### RNA extraction and cDNA synthesis

Harvested cells were disrupted under frozen conditions at 3000 rpm for 1 min, using a Micro-Dismembrator S (Sartorius Stedim Biotech, Göttingen, Germany). Tissue specimens were ground to powder utilising an Eppendorf mortar and pestle system in the presence of liquid Nitrogen. Total RNA was then isolated using a modified RNA Trizol extraction procedure^70^. Briefly, 1 ml Trizol (Invitrogen, San Diego, CA, USA) was added to the tissue or cell material after which chloroform (Sigma-Aldrich) was added to permit for the separation of the RNA from the proteinaceous material. After centrifugation, the aqueous RNA containing phase was transferred to a fresh tube where the RNA was then precipitated out by adding Isopropanol (Sigma-Aldrich). After incubation at RT for 10 min samples were centrifuged at 20000 rpm over-night at 4 °C, upon which RNA pellets were then washed with 75% ethanol (Merck, Billerica MA, U.S.A.) and permitted to dry thoroughly, to prevent alcohol contamination. After drying, total RNA was resuspended in 32 μl RNase free water (Gibco, California, U.S.A.) after which the concentration and purity of the RNA was determined using a NanoDrop^TM^Lite (Thermo Scientific, Waltham, U.S.A.) and quality assessed with a Bioanalyzer 2100 (Agilent Technologies, CA, U.S.A.). After RNA extraction, approximately 1 µg of RNA was reverse transcribed into complementary DNA (cDNA) utilising the QuantiTect Reverse Transcription cDNA Synthesis Kit (Qiagen, Hilden, Germany). CDNA was stored at −20 °C.

### Primer design and optimisation

Candidate reference genes were selected out of a gene library pool, known to be suitable reference genes optimal for the normalization of RNA qRT-qPCR expression studies, which focus on those with a standard deviation of the average amplification threshold cycles (Cq) of less than 1 across 35 in human, rat, mouse cell and tissues^60,71^. Whilst a considerable number of different reference genes are known, for the present study 8 potential reference genes were selected; *TATA-binding protein (TBP)*, *glyceraldehyde 3-phosphate dehydrogenase (GAPDH)*, *RNA 28S ribosomal 4 (RNA28S4)*, *RNA polymerase II subunit e (POLR2e)*, *ribosomal protein lateral stalk subunit P0 (RPLPO)*, *succinate dehydrogenase complex flavoprotein subunit A (SDHA)*, *actin beta (ACTB)*, *ribosomal protein L13a (RPL13a)* (Table 2); as generally these are commonly and stably expressed in most cell and tissue types, taken into consideration with modern qRT-PCR techniques, where more than four reference genes is a rarity for generating accurate relative gene expression data.

**Table 2:** Reference gene primers with amplicon size and annealing temperature ranges for *Rattus norvegicus* and *Homo sapiens*.

Animal	Reference Gene	Accession Number	5′-3′ sequence	3′-5′ sequence	Amplicon Length (bp)	Tm range (°C)
*Rattus noverigus*	*SDHA*	NM_130428.1	GCGGTATGAGACCAGTTATT	CCTGGCAAGGTAAACCAG	239	56–64
	*POLR2e*	BC158787.1	GACCATCAAGGTGTACTGC	CAGCTCCTGCTGTAGAAAC	151	55–64
	*TBP*	BC081939.1	TAACCCAGAAAGTCGAAGAC	CCGTAAGGCATCATTGGA	185	55–64
	*ACTB*	NM_031144.3	AGCTATGAGCTGCCTGA	GGCAGTAATCTCCTTCTGC	243	55–64
	*RPL13a*	NM_173340.2	TTTCTCCGAAAGCGGATG	AGGGATCCCATCCAACA	159	55–62
	*RNA28S4*	NR_145822.1	GCGGCCAAGCGTTCATA	CCTGTCTCACGACGGTCTAA	143	56–65
	*GAPDH*	BC083511.1	CATGGGTGTGAACCATGA	TGTCATGGATGACCTTGG	104	55–63
	*RPLP0*	BC001834.2	CAACCCAGCTCTGGAGA	CAGCTGGCACCTTATTGG	116	55–62
*Homo sapiens*	*SDHA*	NM_001330758.1	CTTCCTTGCCAGGACCTA	GGCGTATCGCTCCATAAAC	117	55–65
	*POLR2e*	J04965.1	CTATCTGGTGACCCAGGA	CTGCAGAAACTGCTCCA	322	55–61
	*TBP*	XX000000.0	CACTTCGTGCCCGAAAC	GCCAGTGTGGACTGTTCT	121	55–63
	*ACTB*	NM_001101.3	CTGCCCTGAGGCACTC	GTGCCAGGGCAGTGAT	197	55–64
	*RPL13a*	NM_012423.3	CTTTCCTCCGCAAGCGG	GTCCGCCAGAAGATGCG	159	55–62
	*RNA28S4*	NR_145822.1	GCGGCCAAGCGTTCATA	CCTGTCTCACGACGGTCTAA	143	56–65
	*GAPDH*	BC083511.1	CATGGGTGTGAACCATGA	TGTCATGGATGACCTTGG	104	55–63
	*RPLP0*	BC001834.2	CAACCCAGCTCTGGAGA	CAGCTGGCACCTTATTGG	116	55–62

The relevant sequences of all candidate reference genes (from *Homo sapiens* and *Rattus novericus*) were obtained from GenBank (http://www.ncbi.nlm.nih.gov/genbank/). Reference gene primer sequences were designed utilising PrimeQuest in conjunction with OligoAnalyzer 3.1 on the IDT website (https://eu.idtdna.com/site) with specificity of the designed primer being confirmed through the use of the Basic Local Alignment Search Tool program on Pubmed Central (https://blast.ncbi.nlm.nih.gov/Blast.cgi).

Primers were optimised using a standard temperature gradient run to determine the optimal range of primer annealing temperatures, utilising 25 ng cDNA (from control cell or tissue samples), with 2x FastStart Essential DNA Green Master (Roche, Basel, Switzerland) and 10 µM of each primer (Table 2) in a final reaction volume of 10 µl. Runs were performed using a LightCycler^®^ 96 thermocycler (Roche, Basel, Swiss), with thermocycling parameters including a pre-incubation of 3 min at 95 °C, followed by a three-step amplification programme of 40 cycles consisting of a denaturation, annealing and extension step set at 95 °C for 10 s, 55 to 65 °C for 15 s and 72 °C for 30 s, respectively. A melt curve was included in each run to confirm amplification of a single product. After PCR amplification wells identified with positive amplicons were purified with the Mini Elute PCR Purification Kit (Qiagen, Crawley, UK), according to the manufacturer’s instructions and analysed, after Sanger sequencing (GATC Biotech, Cologne, Germany) utilising BLAST against the GenBank database to validate primer reference gene sequence amplification specificity.

### Standardisation of cDNA quantity for qRT-PCR

In order to determine optimum cDNA quantity to be used per qRT-PCR reaction in cells and tissue samples a standard curve was utilised. A 2x dilution gradient of cDNA amounts, specifically 40 ng, 20 ng, 10 ng, 5 ng, 2.5 ng and 0 ng, was utilised to generate the standard curve in relation to the Cq. PCR reactions were carried out in 96-well plates in duplicate. The PCR reactions were performed using a qRT-PCR LightCycler^®^ 96 Instrument (Roche, Basel, Swiss), where the total volume per reaction was 10 μl, containing 10 μM of each reference primer (Table 2), the corresponding diluted cDNA and 2x FastStart Essential DNA Green Master (Roche). Standardisation runs were performed using a LightCycler^®^ 96 thermocycler (Roche, Basel, Swiss), with thermocycling parameters possessing a pre-incubation of 3 min at 95 °C, followed by a three step amplification programme of 40 cycles consisting of a denaturation, annealing and extension step set at 95 °C for 10 s, 60 °C for 15 s and 72 °C for 30 s, respectively. A melt curve was included in each run to validate product amplification. Standard curve results were summarised into a table showing the [cDNA] dilution in relation to the Cq value.

### Reference primer expression stability and quantity

All reference primers were tested in hBMSCs and hADSCs undergoing chondrogenic differentiation, normal chondrocytes (with and without treatment) or rat muscle tissue to determine reference primer stability and how many reference primers were needed to generate accurate gene expression data. PCR reactions were carried out in 96-well plates in duplicate utilising a qRT-PCR LightCycler^®^ 96 Instrument (Roche) with a total reaction volume of 10 μl, that contained 10 μM of each reference primer (Table 2), 10 ng of cDNA and 2x FastStart Essential DNA Green Master (Roche). Thermocycling parameters had a pre-incubation step of 3 min at 95 °C, followed by a three step amplification programme of 40 cycles consisting of a denaturation, annealing and extension step set at 95 °C for 10 s, 60 °C for 15 s and 72 °C for 30 s, respectively. A melt curve was included in each run to validate product amplification. Generated data was then inputted into GeNorm (http://medgen.ugent.be/wjvdesomp/genorm/) using the relative quantities based on comparative Cq method^72^. The statistical tools used by GeNorm were used to assess the expression stability of the candidate reference gene using the M-value, which refers to the average pairwise variation between each reference gene and the other reference genes. A gene with M <1.5 is considered as a stable reference gene. Subsequently, the pairwise variation (V-score) was determined which indicates the optimal number of reference genes to use for the cell or tissue type to generate realistic and accurate relative quantitative gene expression data^27,72,73^. The value of Vn/n + 1 under 0.15 indicates that no additional reference genes are required for normalization^72^.

## Data Availability

The necessary algorithmic codes of the program GeNorm are readily available at (http://medgen.ugent.be/wjvdesomp/genorm/). All data, raw and processed, is readily available from the corresponding author on request.
